# FABP5 suppresses colorectal cancer progression via mTOR-mediated autophagy by decreasing FASN expression

**DOI:** 10.7150/ijbs.85285

**Published:** 2023-06-12

**Authors:** Mujie Ye, Chunhua Hu, Tiaotiao Chen, Ping Yu, Jinhao Chen, Feiyu Lu, Lin Xu, Yuan Zhong, Lijun Yan, Jingbao Kan, Jianan Bai, Xiaolin Li, Ye Tian, Qiyun Tang

**Affiliations:** Department of Geriatric Gastroenterology, Neuroendocrine Tumor Center, Jiangsu Province Hospital, The First Affiliated Hospital of Nanjing Medical University, Institute of Neuroendocrine Tumor, Nanjing Medical University, Nanjing, China.

**Keywords:** Colorectal cancer, Lipid metabolism, *FABP5*, * FASN*, Autophagy, N6-methyladenosine, Orlistat

## Abstract

Lipid metabolism plays an important role in the occurrence and development of cancer, in particular, digestive system tumors such as colon cancer. Here, we investigated the role of the fatty acid-binding protein 5 (*FABP5*) in colorectal cancer (CRC). We observed marked down-regulation of *FABP5* in CRC. Data from functional assays revealed inhibitory effects of *FABP5* on cell proliferation, colony formation, migration, invasion as well as tumor growth *in vivo*. In terms of mechanistic insights, *FABP5* interacted with fatty acid synthase (*FASN*) and activated the ubiquitin proteasome pathway, leading to a decrease in *FASN* expression and lipid accumulation, moreover, suppressing *mTOR* signaling and facilitating cell autophagy. Orlistat, a *FASN* inhibitor, exerted anti-cancer effects both *in vivo* and *in vitro*. Furthermore, the upstream RNA demethylase *ALKBH5* positively regulated *FABP5* expression via an m^6^A-independent mechanism. Overall, our collective findings offer valuable insights into the critical role of the *ALKBH5*/*FABP5*/*FASN/mTOR* axis in tumor progression and uncover a potential mechanism linking lipid metabolism to development of CRC, providing novel therapeutic targets for future interventions.

## Introduction

Colorectal cancer (CRC) is the third most common malignancy and second most deadly cancer type worldwide, accounting for estimated 1.9 million new cases and 900,000 deaths in 2020^1^, ^2^. The incidence of CRC is relatively high in developed countries and continues to increase gradually in developing countries^3^, ^4^. Since the early symptoms of CRC are not obvious and colonoscopy is not widely used as a screening tool, timely diagnosis of CRC is a challenge and most cases are detected at the middle and late stages of disease progression. The current clinical treatments for CRC mainly include surgery, chemotherapy and radiotherapy^5^. However, surgical treatment is only suitable for patients with early diagnosis. For patients with advanced CRC, the efficacy of chemotherapy is affected by drug resistance and serious adverse reactions, and the overall treatment effect remains unsatisfactory. As a major threat to human health, CRC management is a significant challenge in the field of cancer research^6^, ^7^. Elucidation of the molecular mechanisms underlying its pathogenesis and identification of effective disease-targeting molecules with minimal side-effects are essential research goals for early diagnosis and treatment.

Lipids are an important component of cellular bio-membranes. In addition to energy storage and metabolism, lipids serve as critical signaling molecules for multiple cellular activities^8^. Regulation of lipid metabolism (such as lipid uptake, synthesis, and hydrolysis) is critical for maintaining cellular homeostasis^9^. Abnormal lipid metabolism is clearly associated with various diseases such as diabetes, cancer, and neurodegenerative disorders^10^. For example, intestinal tumor cells often exhibit abnormal activation of lipid metabolism^11^. Cancer cells in the tumor micro-environment can increase uptake of exogenous lipids or up-regulate endogenous lipogenesis and cholesterol synthesis in order to meet the needs of continuous proliferation during growth and metastasis^12^. Abundant lipids and lipid metabolites are utilized to provide energy and promote rapid tumor cell growth and metastasis. Accordingly, abnormal lipid metabolism is one of the hallmark features of cancer that has attracted considerable research attention in recent years^13^.

Earlier studies have implicated dysregulation of lipid or lipid metabolism-related genes in the occurrence and development of CRC, supporting their value as potential biomarkers for early detection. Therefore, key genes in lipid metabolism could serve as molecular targets for CRC therapy and further elucidation of the underlying mechanisms may have clinical significance^14^, ^15^. Fatty acid binding proteins (FABPs) are intracellular fatty acid carriers that coordinate lipid responses, function in cellular fatty acid utilization, and are highly associated with metabolic and inflammatory pathways. Nine *FABP* genes have been identified in mammals to date, designated *FABP1-7, FABP9* and *FABP12. FABPs* exist in different forms in various tissues, with unique roles and expression patterns in multiple cancer types^16^.

*FABP5* is a relatively low molecular weight lipid chaperone protein involved in regulation of various biological processes, such as fatty acid uptake and transport. *FABP5* is highly expressed in various cancers and closely related to tumor growth, development and metastasis. In an earlier cervical cancer study, *FABP5* was shown to promote epithelial-mesenchymal transition and lymph node metastasis by reprogramming fatty acid (FA) metabolism. Mechanistically, *FABP5* enhanced lipolysis and FA synthesis and activated *NF-κB* signaling, leading to increased levels of intracellular FA, thereby inducing lymph node metastasis^17^. Another study on a mouse model of lung tumor metastasis reported that mice lacking *FABP5* were more prone to metastasis. Further studies revealed that *FABP5* deficiency leads to impaired NK cell maturation in the lung and *FABP5* controls NK cell maturation to regulate lung tumor metastasis^18^. *FABP5* may therefore be of significant clinical value in CRC, since this tumor type is closely related to lipid metabolism.

## Methods

### Human CRC cell lines and tissues

Human CRC cells HCT116 and SW620 were cultured in RPMI-1640 (Biological Industries, Israel) and L15 medium (Fuheng, Shanghai, China), respectively. 293T cells were cultured in Dulbecco's Modified Eagle Medium (DMEM, Biological Industries) supplemented with 10% fetal bovine serum (FBS, Yeasen Biotechnology, Shanghai, China) and 1% penicillin-streptomycin solution (New Cell & Molecular Biotech, Suzhou, China). All cells were grown in a cell incubator under 5% CO_2_ and 37 °C.

### Quantitative real time-polymerase chain reaction (qPCR)

TRIzol (Vazyme, Nanjing, China) was used for extraction of total RNA. Relative cDNA was synthesized using a specific cDNA synthesis kit (Yeasen) under the following conditions: 42 °C for 2 min to digest genomic DNA, followed by 25 °C (5 min), 55 °C (15 min) and 85 °C (5 min) for reverse transcription. Hieff Universal Blue SYBR Green Mix (Yeasen) was used for the qPCR assay (Roche). The PCR protocol was as follows: initial denaturation at 95°C for 5 min, followed by 35 cycles at 95°C (30 s), 58°C (30 s), and 72°C (30 s). GAPDH was used as the internal control. The primers utilized are specified in Supplementary Table 1. Expression of genes was analyzed using GraphPad Prism 6 software.

### Stable transfection of cell lines

*FABP5*, *FASN* and m6A molecules plasmids were purchased from Genomeditech (Shanghai, China) for construction in PLKO1 (knockdown) or PLVX (over-expression) vectors. The short hairpin targets used are presented in Supplementary Table 2. Lentivirus was packaged into 293T cells using PEI MAX transfection reagent (Polysciences, USA). Briefly, 50 µL PEI-MAX transfection reagent was added to 5 µg *FABP5* plasmid along with equivalent amounts of two auxiliary plasmids (PAX2 and PDM2G at a 1:1 ratio), followed by the addition of 1.5 mL serum-free DMEM. After 30 min, plasmids were added to cells in serum-free DMEM. After 6 h, 10% FBS was added to cells and virus collected 48 h later. Stably transfected cells were acquired after virus infection and puromycin screening.

### Cell proliferation assays

Proliferation was detected with CCK-8 (New Cell & Molecular Biotech). Briefly, 5×10^3^ cells were cultured in 100 µL medium in 96-well plates, incubated with 10 µL reagent for 2 h, and analyzed using a microplate reader at a wavelength of 450 nm. For the colony formation assay, 1 × 10^4^ cells were seeded in a 6-well plate and cultured for one week, followed by fixing with 4% paraformaldehyde and staining with 0.25% crystal violet. The 5-ethynyl-2′-deoxyuridine (EdU) assay was conducted as described previously. Briefly, a 1:1000 dilution of EdU was added to 96-well cell plates. After 2 h, cells were subjected to fixing, EdU staining, Hoechst 33342 staining, and washing (PBS), and images obtained under a microscope.

### Cell migration and invasion assays

For the cell migration and invasion assays, 8 μm micropore inserts in 24-well cell culture plates were used. For cell migration experiments, 2 × 10^5^ cells were seeded into upper wells without FBS. For cell invasion experiments, 4 × 10^5^ cells were seeded into upper wells coated with 50 μL diluted matrigel (Becton, Dickinson) without FBS. In transwell assay, 30% FBS was added to lower wells. Wells were fixed with 4% paraformaldehyde for 10 min and stained with 0.25% crystal violet for 20 min.

### Western blot

Cellular proteins were extracted using NP40 lysis buffer (Beyotime, Nantong, China) on ice for 30 min. After measuring protein concentrations using the Bradford method (Beyotime), samples were boiled for 10 min at 100 ℃ in 1× SDS protein loading buffer (Yeasen). Following standard electrophoresis and transfer, the membrane (Millipore, USA) was blocked with skimmed milk (8%) for 60 min. Next, primary antibodies (listed in Supplementary Table 3) were added to the membrane on a glass plate for overnight incubation at 4 °C. After washing three to four times with Tris-buffered saline with Tween 20(TBST) buffer, samples were incubated with the appropriate secondary antibodies for 1 h at room temperature. After washing with TBST for three times, signals of bands were detected using the Enhanced Chemiluminescent Reagent kit (New Cell & Molecular Biotech). Image J software was used to quantitative gray value of the bands.

### Co-immunocoprecipitation

RIPA lysis (1 ml) were added to 10 cm dish with cells and proteins were extracted following western blot methods. After incubation with 2 µg antibody for 2 h at 4°C, 30 µl protein A/G magnetic beads (Beyotime) was added and inverted overnight. Next day, samples were washed thoroughly with RIPA lysis buffer (Beyotime) three times, incubated in 30 µl of 2×SDS-PAGE sample loading buffer (Beyotime) and boiled at 100°C for 10 min for subsequent western blot experiments.

### Animal assays

For generation of animal models, 1 × 10^6^ CRC cells from each group were subcutaneously injected into the flanks of 4-6 week old BALB/c nude mice in 100 µL PBS. To ascertain the effect of orlistat on tumor growth *in vivo*, orlistat in oil (10 mg/kg/day) was intragastrically administered following transplantation of cells into mice. After three weeks, mice were sacrificed and tumors collected to evaluate volumes and weights. Subsequently, tumor tissues were fixed and slides prepared for immunohistochemistry of *FABP5* and *FASN*. The immunohistochemistry procedure was conducted as described previously^19^. Animal studies were approved by Institutional Animal Care and Use Committee of Nanjing Medical University.

### Statistical analysis

Results are presented as mean ±SD and analyzed using GraphPad Prism 6.0 software. Student's t-test was used to assess significant differences in two-group comparisons. P values < 0.05 were considered significant. All *in vitro* assays were independently repeated at least three times.

## Results

### *FABP5* is down-regulated in CRC

**Figure 1:**
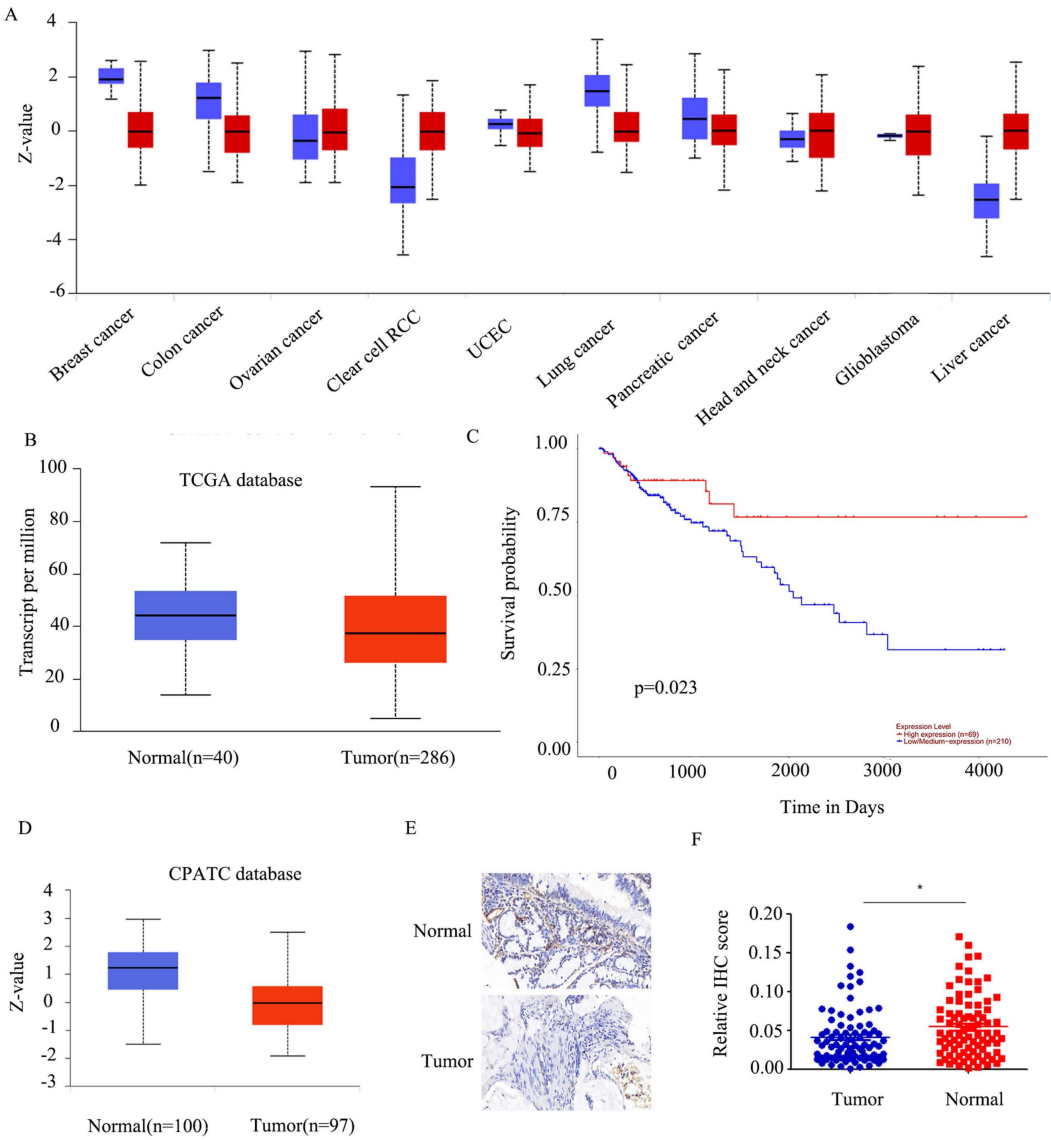
***FABP5* is silenced in CRC.** A) *FABP5* protein levels in ten cancers from the CPATC database. (B) *FABP5* was down-regulated in CRC compared to normal tissues in the TCGA database(p=2.3e-1). (C) Low expression of *FABP5* was associated with shorter survival probability in CRC cases from the TCGA database. (D) Protein expression of *FABP5* in CRC from the CPATC database(p=7.73e-13). (E) Representative immunohistochemical staining of *FABP5* in CRC tissues from patients, magnification: ×73. (F) Relative average optical density values in 90 CRC and paired normal tissues. (*P < 0.05)

To establish the precise function of *FABP5* in cancer, we investigated its expression patterns in various tumor types. *FABP5* was over-expressed in renal clear cell carcinoma and liver cancer and, conversely, down-regulated in lung, breast and colon cancer (Figure 1A). Moreover, the *FABP5* mRNA level was lower, but not to a significant extent, in tumor than control tissues (Figure 1B). Data from survival analyses indicated better prognosis of patients with higher *FABP5* levels (Figure 1C).

Consistent with findings from the CPATC database (Figure 1D), our results showed down-regulation of *FABP5* in CRC. As few reports have explored the function of *FABP5* in CRC, we initially established a tumor microarray using 90 CRC and peri-tumorous tissues. And the results indicated FABP5 significantly down-regulated in CRC (Figure 1E-1F).

### Over-expression of *FABP5* is associated with reduced CRC cell proliferation, migration and invasion

**Figure 2:**
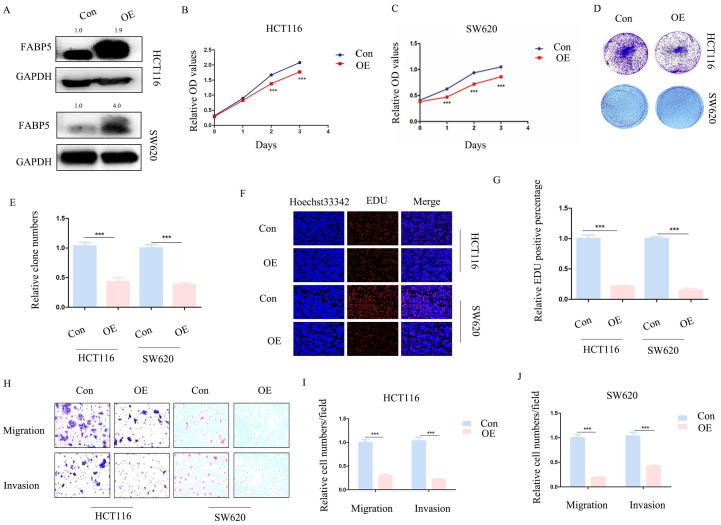
** Over-expression of *FABP5* suppresses cell proliferation, migration and invasion.** Western blot analysis of alterations in protein levels of *FABP5* in over-expressing and control CRC cells. (B, C) Over-expression of *FABP5* inhibited proliferation of HCT116 (B) and SW620 (C) cells, determined via the CCK8 assay. (D, E) Up-regulation of *FABP5* induced a decrease in the number of colonies. (F, G) EdU assay showed suppression of proliferation in both cell types under conditions of *FABP5* over-expression, magnification: ×200. (H-J) *FABP5* suppressed migration and invasion of HCT116 and SW620 cells, magnification: ×100. (**P < 0.01, ***P < 0.001)

To ascertain whether *FABP5* plays a tumor suppressor role in CRC, stable *FABP5* over-expressing HCT116 and SW620 cell lines were generated via stable transfection. Western blot results validated the efficiency of *FABP5* over-expression (Figure 2A). In CCK-8 and EdU assays, up-regulation of *FABP5* was concomitant with suppression of cell proliferation (Figure 2B-2C; 2F-2G). Moreover, data from the clone formation assay showed decreased clone numbers in *FABP5*-expressing HCT116 and SW620 cells (Figure 2D-2E). In the transwell assay, up-regulation of *FABP5* suppressed cell migration and invasion (Figure 2H-2J). Overall, FABP5 functioned as anti-cancer in CRC.

### Knockdown of *FABP5* promotes CRC cell proliferation, migration and invasion

**Figure 3:**
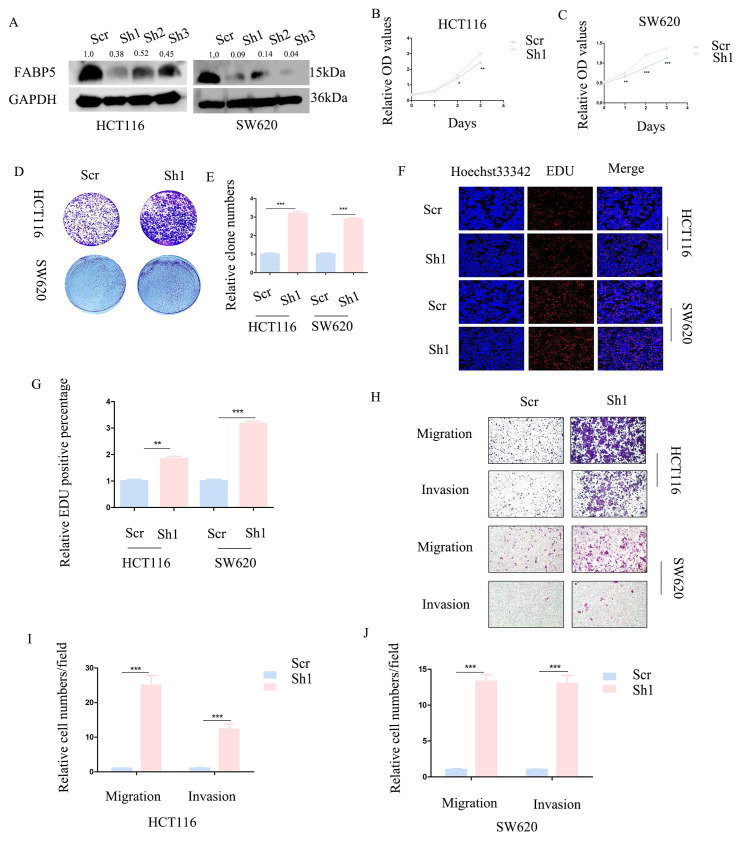
** Knockdown of *FABP5* promotes malignant biological behaviors of CRC cells.** (A) Western blot analysis of the protein levels of *FABP5* in knockdown and control CRC cells. (B, C) CCK-8 data showing that silencing of *FABP5* promoted proliferation of HCT116 (B) and SW620 (C) cells. (D, E) Down-regulation of *FABP5* induced an increase in the number of colonies. (F, G) Down-regulation of *FABP5* accelerated proliferation of HCT116 and SW620 cells, as observed with the EdU assay, magnification: ×200. (H-J) Silence of *FABP5* facilitated cell migration and invasion of both cell types, magnification: × 100. (*P < 0.05, **P < 0.01, ***P < 0.001).

To further establish the anti-cancer function of *FABP5* in CRC, HCT116 and SW620 cell lines with stable knockdown of *FABP5* were generated. Western blot analysis validated the efficiency of *FABP5* knockdown (Figure 3A). In CCK-8 and EdU experiments, down-regulation of *FABP5* promoted cell proliferation (Figures 3B-3C, 3F-3G). Notably, suppression of *FABP5* increased the clone numbers of HCT116 and SW620 in the clone formation assay (Figure 3D-3E). Data from the transwell assay showed that *FABP5* silencing facilitated migration and invasion of HCT116 and SW620 cells (Figure 3H-3J), supporting a tumor suppressor role of *FABP5* in CRC cells.

### *FABP5* interacts with *FASN* and promotes its ubiquitin proteasome pathway

**Figure 4:**
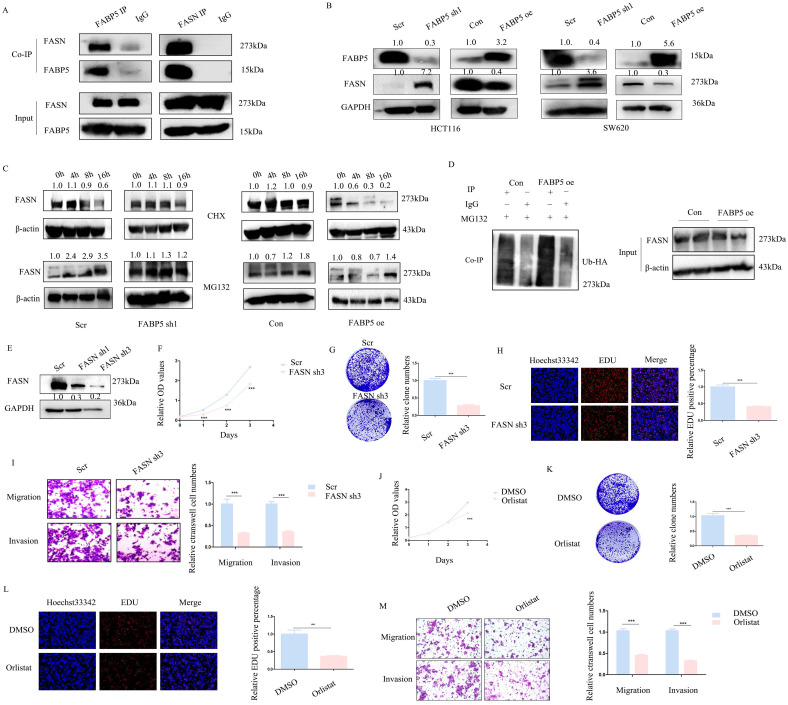
***FABP5* functions as a tumor suppressor via interacting with *FASN.*** (A) Co-IP of *FABP5* and *FASN* in HCT116 cells. (B) Western blot analysis of *FASN* levels in *FABP5* over-expressing and silenced HCT116 and SW620 cells. (C) Western blot analysis of *FASN* in *FABP5* over-expressing and depleted HCT116 cells subjected to CHX (10 µmol/L) and MG132 (10 μmol/L) treatment. (D) Co-IP analysis of *FASN* and ubiquitin in *FABP5* over-expression HCT116 cells treated with MG132 (10 μmol/L) for 6 h. (E) Western blot showing *FASN* knockdown efficiency in HCT116 cells. (F) Silencing of *FASN* inhibited proliferation of HCT116 cells. (G, H) Down-regulation of *FASN* induced a decrease in colony number and proliferation of cells. (I) Silencing of *FASN* suppressed cell migration and invasion, magnification: ×100. (J-L) Cell proliferation was significantly decreased upon treatment with orlistat (50 µmol/L for 24 h), as observed with CCK-8 (J), colony formation (K) and EdU assays (L), magnification: ×200. (M) Orlistat inhibited migration and invasion of HCT116 cells, magnification: ×100. (**P < 0.01, ***P < 0.001)

With the aid of combined immunoprecipitation and mass spectrometry analyses, 395 proteins interacting with *FABP5* were identified. Among these proteins, *FASN* attracted us attention, which was a fatty acid synthase. Subsequent Co-IP experiments confirmed interactions of *FABP5* with *FASN* (Figure 4A). Notably, knockdown of *FABP5* led to an increase in *FASN* expression and, conversely, over-expression of *FABP5* induced a decrease in *FASN* (Figure 4B). After treatment with CHX, the stability of *FASN* was increased in *FABP5* knockdown and decreased in *FABP5* over-expression groups. Moreover, the *FASN* level was markedly increased upon MG132 treatment (Figure 4C). Co-IP assay of *FASN* and ubiquitin consistently showed that over-expression of *FABP5* led to an increase in ubiquitin combined with *FASN* (Figure 4D). To further resolve its function in CRC, *FASN* was inhibited via shRNA or treatment with orlistat. Under conditions of knockdown of *FASN* (Figure 4E, Supplementary Figure 1A), cell proliferation (Figure 4F, 4H; Supplementary Figure 1B, 1E-1F), clone formation (Figure 4G, Supplementary Figure 1C-1D), cell migration and invasion (Figure 4I; Supplementary Figure 1G-1H) were inhibited. Similar phenomena were observed in the orlistat treatment group (Figure 4J-4M; Supplementary Figure 1I-1Q). The collective results clearly indicate that *FABP5* regulates *FASN* via stimulation of its ubiquitin proteasome pathway.

### Knockdown or inhibition of *FASN* suppresses malignant biological behaviors activated by down-regulation of *FABP5*

**Figure 5:**
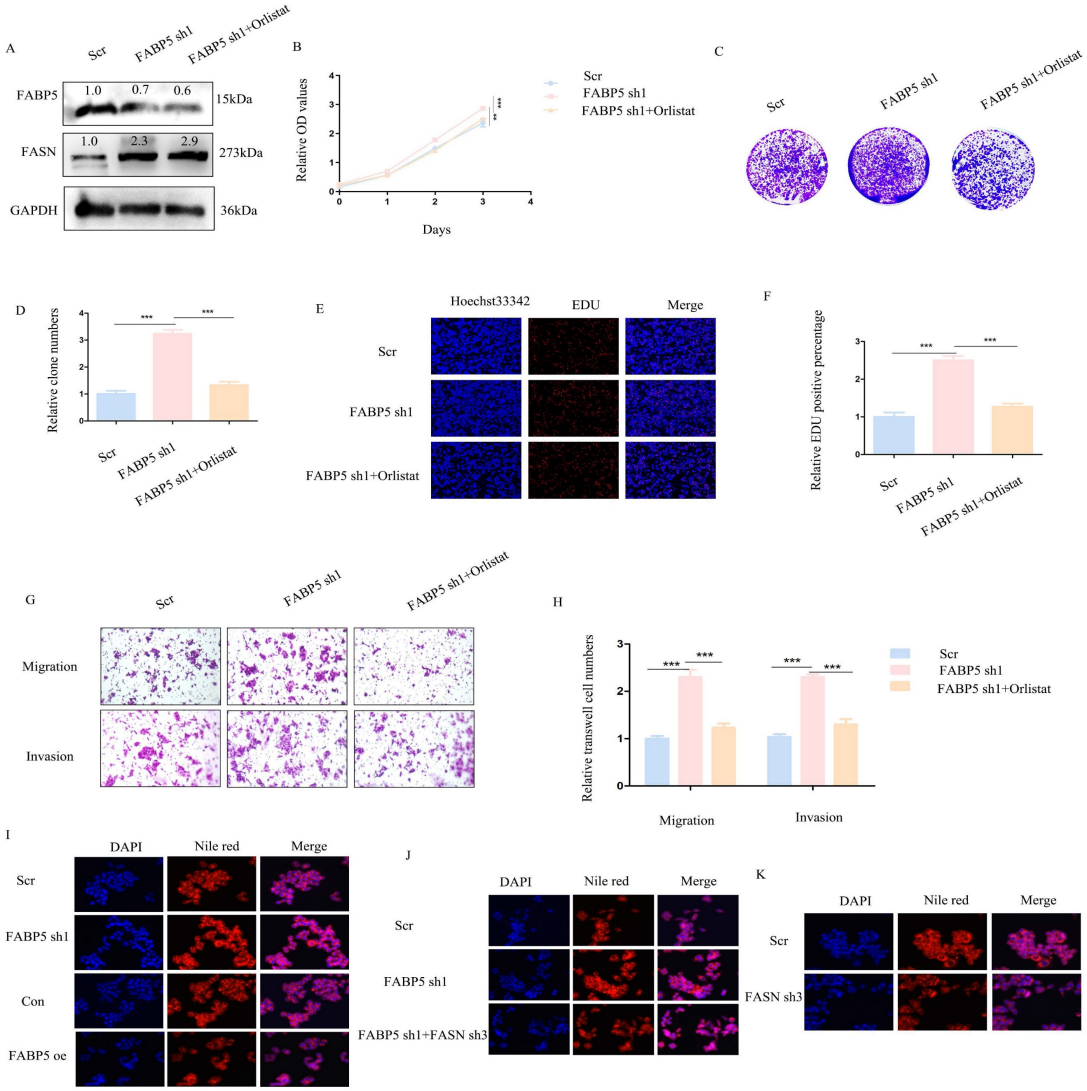
** Orlistat restores CRC cell malignant behaviors induced by *FABP5* down-regulation.** (A) Western blot showing *FABP5* and *FASN* protein levels under conditions of *FABP5* silencing and 50 μmol/L orlistat treatment for 24 h. (B-F) CCK-8, colony formation and EdU assays (Magnification: ×200) showing cell proliferation under conditions of *FABP5* knockdown and orlistat treatment. (G, H) Orlistat reverses CRC cell migration and invasion (Magnification: ×100) induced by *FABP5* silencing. (I-K) Nile red staining (Magnification: ×200) in *FABP5*/*FASN* altered cells. (***P < 0.001)

Above results indicated down-regulation of *FABP5* promotes malignant biological behaviors in CRC. To explore whether oncogenic activity is mediated by up-regulation of *FASN*, knockdown of *FASN* or orlistat treatment in *FABP5* down-regulation stably transfected cells was performed. Firstly, efficiency of knockdown was detected via western blot (Figure 5A; Supplementary Figure 2A). CCK-8, colony formation and EdU experiments revealed that down-regulation of *FASN* or inhibition of its activity led to suppression of cell proliferation promoted by knockdown of *FABP5* (Figure 5B-5F; Supplementary Figure 2B-2F). In the transwell assay, silencing or inhibition of *FASN* reversed the increase in migration and invasion induced by down-regulation of *FABP5* (Figure 5G-5H; Supplementary Figure 2G-2H). Nile red staining demonstrated that over-expression of *FABP5* decreased while its knockdown increased lipid accumulation (Figure 5I). *FASN* depletion resulted in attenuation of lipid accumulation induced by *FABP5* silencing, highlighting a key role of *FASN* in *FABP5*-regulated malignant behaviors (Figure 5J). Moreover, sole knockdown of *FASN* in wild-type CRC cells consistently led to a decrease in lipid accumulation (Figure 5K). These results suggest that oncogenic progression initiated by down-regulation of *FABP5* is restored by *FASN* inhibition in CRC cells.

### *FABP5* promotes autophagy via inactivation of the *mTOR* pathway mediated by *FASN*

**Figure 6:**
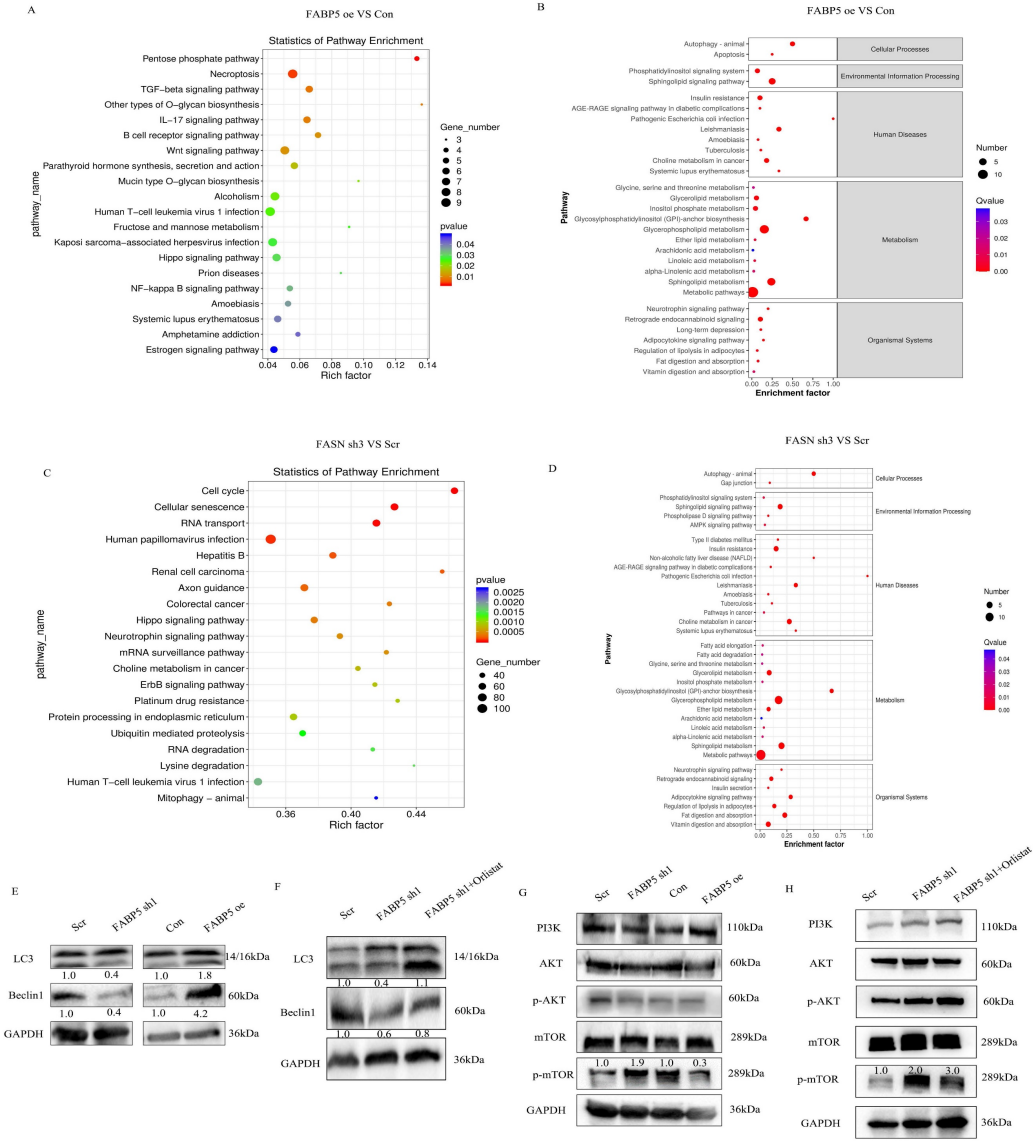
***FABP5* interacts with *FASN* to promote cell autophagy via *mTOR.*** (A-D) Enrichment analysis of transcriptome and lipid metabolomes. (E, F) Western blot analysis of LC3 and Beclin1 in *FABP5* over-expression or down-regulation and *FABP5* down-regulation+orlistat treatment groups. (G, H) Western blot analysis of PI3K/AKT/*mTOR* in *FABP5* over-expression or down-regulation and *FABP5* +orlistat treatment groups.

To explore the mechanisms underlying the tumor suppressor role of *FABP5* in CRC, RNA-seq and lipid-omics were performed in *FABP5* over-expression and *FASN* knockdown along with the respective control groups. Multiple genes associated with numerous biological processes and signaling pathways were dysregulated, including TGF-β, Hippo, Wnt, *NF-κB* and *mTOR*, as observed via RNA-seq (Figure 6A, 6C; Supplementary Figure 3A). Lipid-omic analyses further revealed that *FABP5* and *FASN* regulated autophagy through effects on lipid metabolism (Figure 6B, 6D; Supplementary Figure 3B). Western blot results confirmed that up-regulation of *FABP5* promoted while knockdown of *FABP5* inhibited autophagy (Figure 6E). Moreover, silence of FASN or treatment with orlistat rescued inhibition of autophagy induced by *FABP5* silencing (Figure 6F, Supplementary Figure 2J, Supplementary Figure 3C, 3E). Similar to this finding, over-expression of *FABP5* suppressed, while knockdown of *FABP5* activated, the *mTOR* pathway (Figure 6G). Both silencing of *FASN* and treatment with *FASN* inhibitor inactivated the *mTOR* pathway (Figure 6H; Supplementary Figure 2I; Supplementary Figure 3D, 3F), supporting a tumor suppressor role of *FABP5* via inhibition of *mTOR* through *FASN*.

### Over-expression of *FABP5* and knockdown of *FASN* inhibits tumor growth *in vivo*

**Figure 7:**
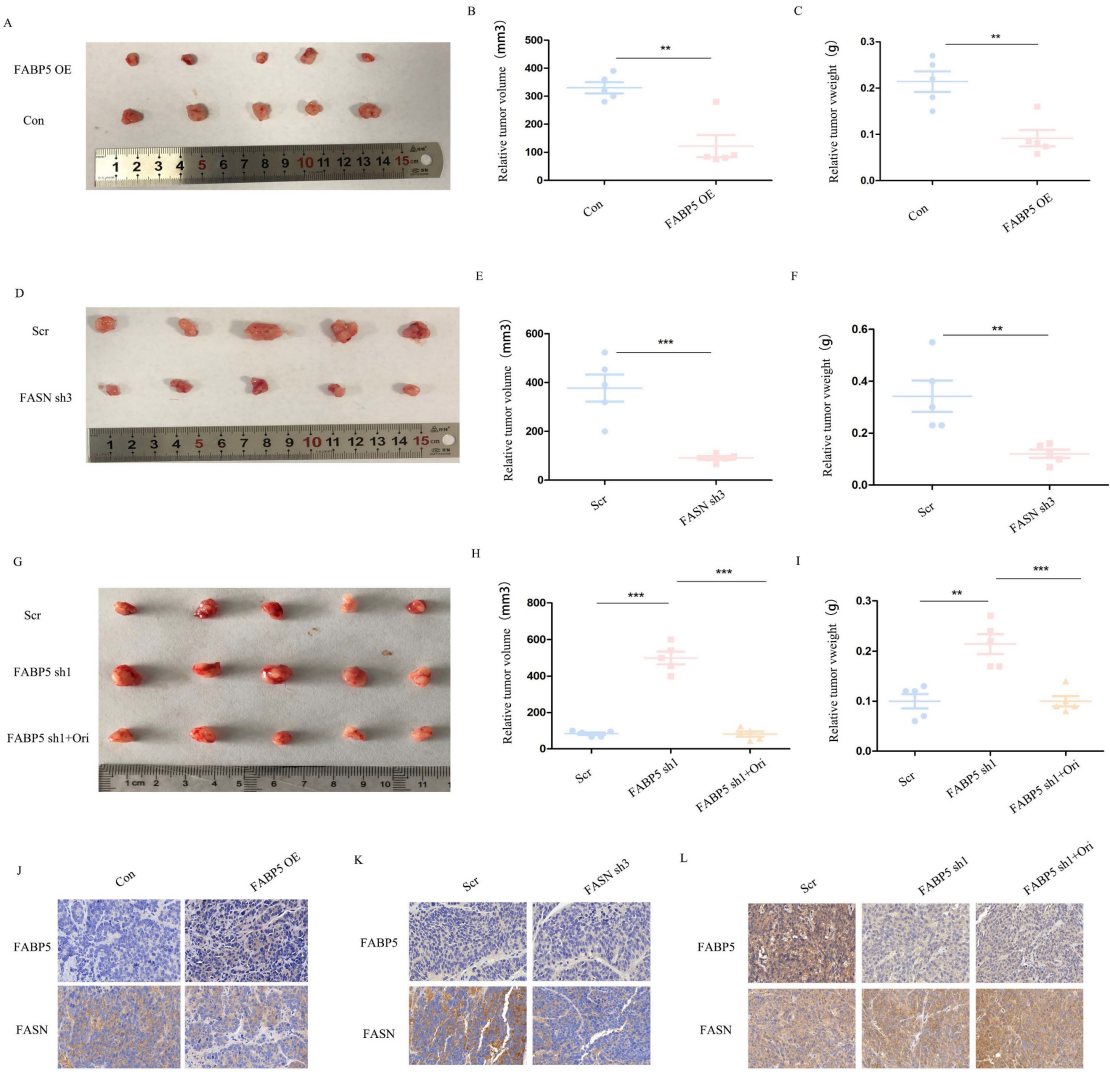
***FABP5* suppresses tumor growth via regulation of *FASN in vivo***. (A-C) Primary tumor samples obtained from mice subcutaneously injected with HCT116 cells transfected with *FABP5* over-expression and control cell groups (A). Relative tumor volumes (B) and weights (C) at the endpoint (n = 5). (D-F) Primary tumor samples were obtained from mice subcutaneously injected with HCT116 cells transfected with *FASN* knockdown and control group (D). Relative tumor volumes (E) and weights (F) measured at the endpoint (n = 5). (G-H) Primary tumor samples obtained from mice subcutaneously injected with HCT116 cells with *FABP5* silencing, *FABP5* silencing plus orlistat treatment, and control groups (G). Relative tumor volumes (H) and weights (I) measured at the endpoint (n = 5). (J-L) Representative immunohistochemistry images showing expression of *FABP5* and *FASN* in xenograft tumor tissues, magnification: ×73. (**P < 0.01, ***P < 0.001)

We further examined the roles of *FABP5* and *FASN in vivo* with the aid of tumor xenograft models. To this end, SW620 cells transfected with *FABP5* over-expression or *FASN* knockdown vector, *FABP5* knockdown vector plus orlistat and control vector were implanted in nude mice. Notably, the weights and volumes of tumors were decreased in mouse xenografts injected with SW620 cells bearing *FABP5* over-expression and *FASN* knockdown vectors (Figure 7A-7F). Conversely, SW620 cells with stable *FABP5* knockdown showed accelerated tumor growth compared with the control group, which was suppressed by orlistat (Figure 7G-7I). Immunohistochemical staining of *FABP5* and *FASN* revealed FABP5 negatively regulated FASN expression in vivo (Figure 7J-7L). In summary, our collective results support an essential role of *FABP5* in *FASN*-mediated CRC progression, both *in vitro* and *in vivo*.

### *ALKBH5* regulates *FABP5* in an m6A-independent manner

**Figure 8:**
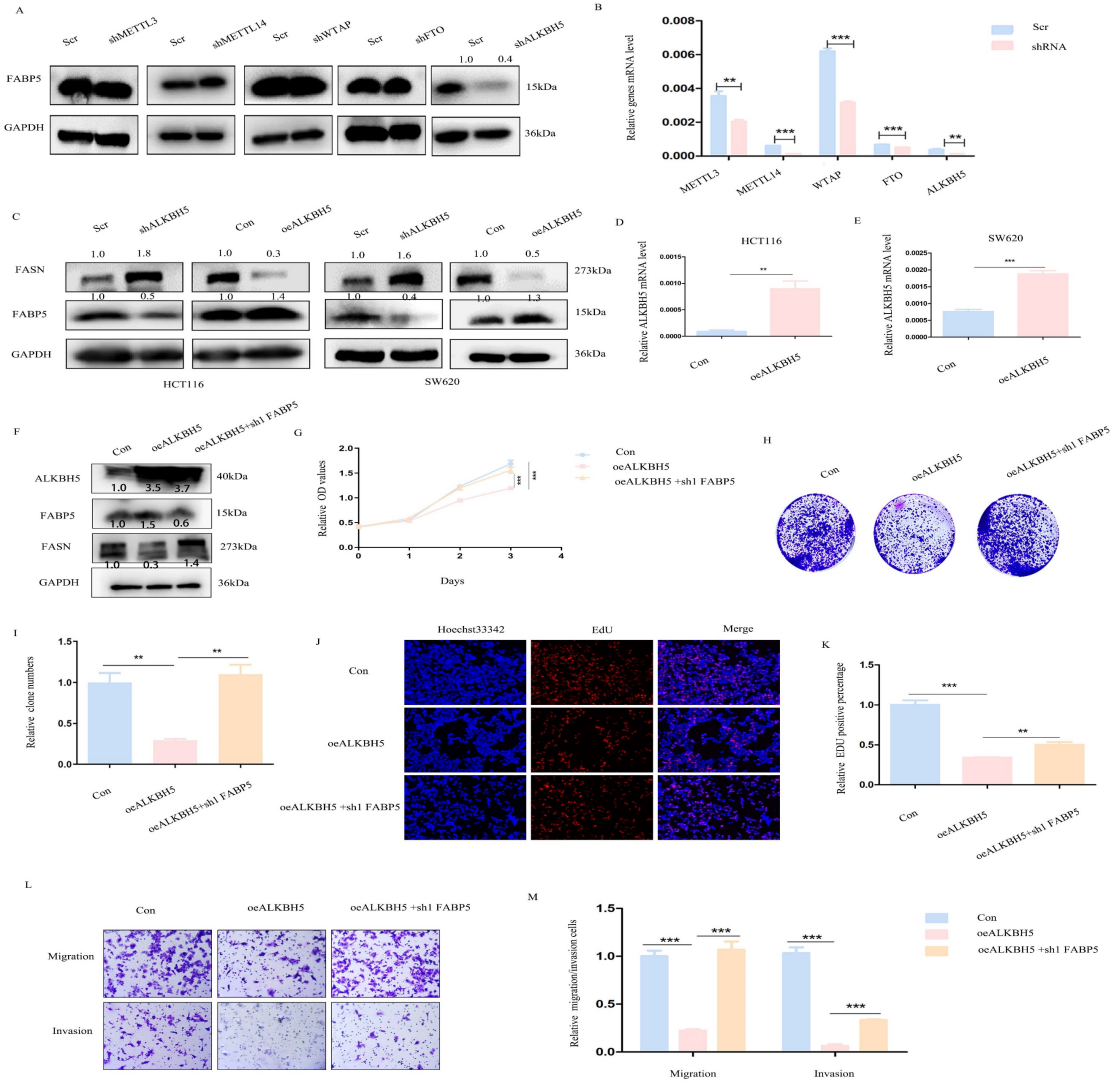
***ALKBH5* positively regulates *FABP5* to exert anti-cancer effects.** (A) Western blots showing positive regulation of *FABP5* by *ALKBH5* via down-regulation of m6A writers (METTL3, METTL14, WTAP) and erasers (FTO, *ALKBH5*). (B) Knockdown efficiency of m6A molecules assessed via RT-PCR. (C) Western blot showing *FABP5* and *FASN* expression under conditions of *ALKBH5* up-regulation and down-regulation. (D, E) Efficiency of *ALKBH5* over-expression assessed via RT-PCR. (F) *ALKBH5*, *FABP5* and *FASN* protein levels evaluated under conditions of *ALKBH5* over-expression, *ALKBH5* over-expression with *FABP5* knockdown and control in HCT116 cells. (G-M) *FABP5* knockdown reversed the decrease in cell proliferation, colony formation, migration and invasion induced by *ALKBH5* in CCK-8 (G), colony formation (H, I), EdU (J, K; Magnification: ×200) and transwell assays (L, M; Magnification: ×100). (**P < 0.01, ***P < 0.001)

Next, we focused on the mechanisms underlying down-regulation of *FABP5* in CRC. The m6A prediction server, SRAMP, revealed abundant m6A modification sites in *FABP5*. Upon knockdown of m6A writers (*METTL3, METTL14, WTAP*) and erasers (*FTO* and *ALKBH5*), positive regulation of *FABP5* was observed in the absence of *ALKBH5* (Figure 8A, 8B). Furthermore, *ALKBH5* negatively modulated the *FASN* level (Figure 8C-8E). To explore whether *ALKBH5* inhibited CRC via *FABP5*, we additionally depleted *FABP5* in *ALKBH5* over-expressing cells (Figure 8F). The anti-cancer effects of *ALKBH5* (including suppression of cell proliferation, migration and invasion) were partly restored by silencing of *FABP5* which indicated *FABP5* could be downstream gene of *ALKBH5*(Figure 8G-8M).

## Discussion

Lipid metabolism plays an important role in tumorigenesis and development^20^, ^21^. During malignant progression, the availability of nutrients in the tumor micro-environment constantly changes and tumor cells utilize the lipid metabolism to sustain rapid proliferation and metastasis^22^, ^23^. Disruption of lipid metabolism is an upcoming novel therapeutic strategy. Therefore, clarification of the molecular mechanisms underlying the association of CRC with lipid metabolism may provide effective therapeutic targets. In this study, we comprehensively explored the roles of *FABP5* and *FASN* in CRC. Our data showed that *FABP5* is down-regulated in CRC tissues and functions as a tumor suppressor via interactions with *FASN*. Furthermore, *FABP5* is regulated upstream by *ALKBH5*, a m6A demethylase. Importantly, knockdown or inhibition of *FASN* dramatically suppressed tumor progression, both *in vivo* and *in vitro*.

As fatty acids play a critical role in lipid metabolism, *FABP5* and *FASN* have been widely studied in numerous diseases, particularly cancer^24^-^26^. *FABP5* is up-regulated and promotes tumor development in gastric cancer, breast cancer, cervical cancer, prostate cancer and hepatocellular carcinoma^25^. *FABP5* silencing has been shown to decrease cell proliferation and lead to cell cycle arrest in gastric cancer.^27^ In breast cancer, *FABP5* activates *VEGF* and up-regulates* EGFR* expression, in turn, increasing *PPAR* to promote cancer progression^28^. Similar phenomena are reported in prostate cancer, showing that *FABP5* activates* PPARγ* and up-regulates *VEGF*^29^. In cervical cancer, *FABP5* reprograms lipid metabolism and facilitates epithelial-mesenchymal transition and lymph node metastasis via activating the *NF-κB* pathway^17^. Moreover, *FABP5* is reported to accelerate tumor metastasis via regulation of *MMP*9 and *MMP*2^30^. However, opposite patterns are observed in skin tumors, whereby *FABP5* suppresses skin tumorigenesis through regulation of the *IFN/p53/SOX2* pathway^31^. These conflicting findings suggest complex roles of *FABP5* in cancer development. The function of *FABP5* in CRC is not well understood at present.

Based on immunoprecipitation and mass spectrometry analyses, *FASN* was identified as an interacting protein with *FABP5*. Furthermore, our results suggested that *FABP5* destabilizes *FASN* via activating its ubiquitin-proteasome pathway. Orlistat, a potent and long-acting specific gastrointestinal fatty acid synthase inhibitor, is the only FDA-approved over-the-counter (OTC) diet medication available worldwide. Jin and co-workers reported that orlistat could alleviate colon cancer induced by western diet-associated colitis via suppression of *STAT3* and *NF-κB* signaling pathways^32^. The group of Zhou and colleagues demonstrated that orlistat decreases *GPX4* and increases lipid peroxidation, promoting ferroptosis in lung cancer cells^33^. In the current investigation, knockdown or inhibition of *FASN* significantly inhibited proliferation, migration and invasion of CRC. Moreover, orlistat reversed cell malignant behaviors induced by *FABP5* knockdown, clearly indicating that cancer progression is induced by *FABP5* down-regulation through effects on *FASN*. Autophagy is considered an important biological process in tumor development. Classical autophagy is mediated by *mTOR* signaling^34^, ^35^. Data from the present study showed that over-expression of *FABP5* and concomitant down-regulation of *FASN* result in inactivation of the *mTOR* pathway, and consequently, increased autophagy. However, the effect of *FABP5*/*FASN* on *mTOR* is not mediated by the known upstream PI3K/AKT pathway.

*ALKBH5* is a type of RNA demethylase (also known as eraser), which is down-regulated in CRC. We detected numerous m6A modification sites in *FABP5* mRNA in CRC. Data from knockdown studies on m6A methylase and demethylase showed positive regulation of *FABP5* expression by *ALKBH5*. Moreover, *FABP5* silencing reversed the anti-cancer activity of *ALKBH5*. However, a separate RNA sequencing study (MeRIP-seq) by our group showed no regulatory effects of *ALKBH5* on *FABP5*. Accordingly, we assume that *ALKBH5* regulates *FABP5* in a non-m6A-dependent manner.

In summary, the present study shows that *FABP5* is down-regulated and *FASN* is induced via suppression of the ubiquitin-proteasome pathway, leading to regulation of autophagy via *mTOR* signaling in CRC. Under conditions of *FABP5* deficiency, lipid accumulation is elevated, ultimately accelerating CRC progression. Our study supports a crucial role of the *ALKBH5*/*FABP5*/*FASN/mTOR* axis in regulation of CRC progression and offer promising therapeutic targets for management of the disease.

## Supplementary Material

Supplementary figures and tables.

## Abbreviations

CRC: Colorectal cancer
FABP5: Fatty acid-binding protein 5
FASN: Fatty acid synthase
Co-IP: Co-Immunoprecipitation
CCK8: Cell counting kit-8
DMEM: Dulbecco's modified eagle medium
FBS: Fetal bovine serum
RT-qPCR: Quantitative real-time polymerase chain reaction
GAPDH: Glyceraldehyde-3-phosphate dehydrogenase
EdU: 5-Ethynyl-2'-deoxyuridine
