# Supplementary material for: FABP5 suppresses colorectal cancer progression via mTOR-mediated autophagy by decreasing FASN expression
Source: Int J Biol Sci. 2023 Jun 12;19(10):3115–27. doi: 10.7150/ijbs.85285 (PMC10321282; doi:10.7150/ijbs.85285)

Supplementary Table1 Primers of genes

Gene names	Sequence(5'-3')
FABP5 forward	TGAAGGAGCTAGGAGTGGGAA
FABP5 reverse	TGCACCATCTGTAAAGTTGCAG
FASN forward	AAGGACCTGTCTAGGTTTGATGC
FASN reverse	TGGCTTCATAGGTGACTTCCA
METTL3 forward	TTGTCTCCAACCTTCCGTAGT
METTL3 reverse	CCAGATCAGAGAGGTGGTGTAG
METTL14 forward	CGCCTCCTCCCAAATCTAAA
METTL14 reverse	CCTCTAAAGCCACCTCTTTCTC
WTAP forward	TTGTAATGCGACTAGCAACCAA
WTAP reverse	GCTGGGTCTACCATTGTTGATCT
FTO forward	GCTGCTTATTTCGGGACCTG
FTO reverse	AGCCTGGATTACCAATGAGGA
ALKBH5 forward	CGGCGAAGGCTACACTTACG
ALKBH5 reverse	CCACCAGCTTTTGGATCACCA
GAPDH forward	GGAGCGAGATCCCTCCAAAAT
GAPDH reverse	GGCTGTTGTCATACTTCTCATGG

Supplementary Table2 Short hairpin targets

Gene names	Target sequence(5'-3')
FABP5 sh1	GGCGCCTGGTGGACAGCAAAG
FABP5 sh2	GCAATGGCCAAGCCAGATTGT
FABP5 sh3	GTCTGCAACTTTACAGATGGT
FASN sh1	CCTACTGGATGCGTTCTTCAA
FASN sh2	GCTACGACTACGGCCCTCATT
FASN sh3	CATGGAGCGTATCTGTGAGAA
METTL3 sh	GCCAAGGAACAATCCATTGTT
METTL14 sh	GAAGACGCCTTCATCTATTTG
WTAP sh	GGCAAGTACACAGATCTTAAC
FTO sh	TCACCAAGGAGACTGCTATTT
ALKBH5 sh	CCACCCAGCTATGCTTCAGAT

SupplementaryTable3 Antibody information

Antibody	Company	Catalogue	Dilution ratio
GAPDH	Proteintech	60004-1-Ig	1：5000
β-Actin	Proteintech	60008-1-Ig	1：5000
LC3	CST	12741S	1：1000
Beclin1	CST	3495S	1：1000
mTOR	CST	2983S	1：1000
p-mTOR2481	CST	2974S	1：1000
PI3K	Proteintech	20584-1-AP	1：1000
AKT	CST	9272S	1：1000
p-AKT ser473	CST	4060T	1：1000
FABP5	CST	39926T	1：1000
FASN	CST	3180S	1：1000
ALKBH5	Abcam	ab195377	1：1000
HA	Proteintech	51064-2-AP	1：1000
Goat Anti-Mouse IgG	CWBIO	CW0102S	1：5000
Goat Anti-Rabbit IgG	CWBIO	CW0103S	1：2000

Supplementary Figure 1 (A) Western blot showing knockdown efficiency of *FASN* in SW620 cells. (B–H) CCK-8 (B), colony formation (C, D), EdU (E, F) and transwell (G, H) assays showing proliferation and migration/invasion abilities of SW620 cells transfected with scramble and *FASN* knockdown plasmids. (I, J) IC50 values of orlistat in HCT116 (I) and SW620 (J) cells determined after 24 h treatment via CCK-8 assay. (K–Q) CCK-8 (K), colony formation (L, M), EdU (N, O) and transwell (P, Q) assays showing proliferation and migration/invasion abilities of cells treated with orlistat for 24 h. Magnification: EdU assay, ×200; transwell, ×100 (**P < 0.01, ***P < 0.001)

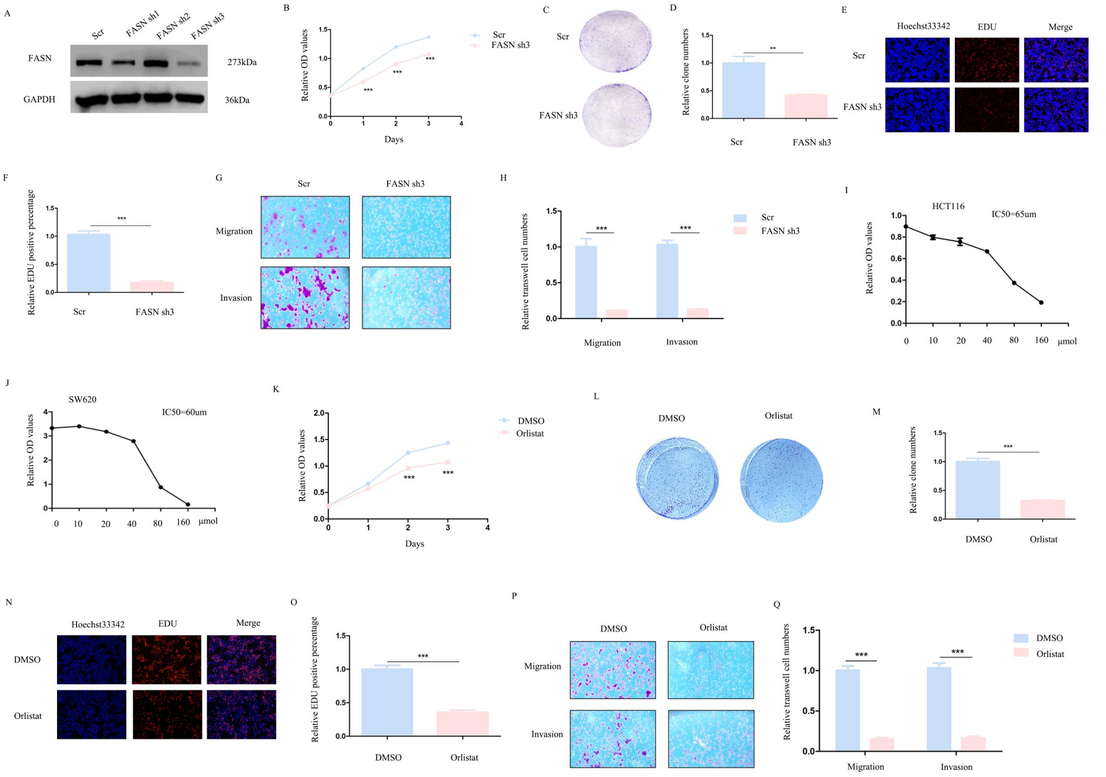

Supplementary Figure 2 (A) Western blot analysis of *FABP5* and *FASN* protein levels in *FABP5* knockdown and *FASN* reversed cells. (B) CCK-8 (B), colony formation (C, D), EdU (E, F) and transwell (G, H) assays showing proliferation and migration/invasion abilities of HCT116 cells transfected with scramble, *FABP5* knockdown and *FABP5&FASN* knockdown plasmids. (I, G) Detection of PI3K/AKT/*mTOR* and LC3/Beclin1 levels via western blot in *FABP5* knockdown and *FASN* reversed cells. Magnification: EdU assay, ×200; transwell, ×100 (**P < 0.01, ***P < 0.001).

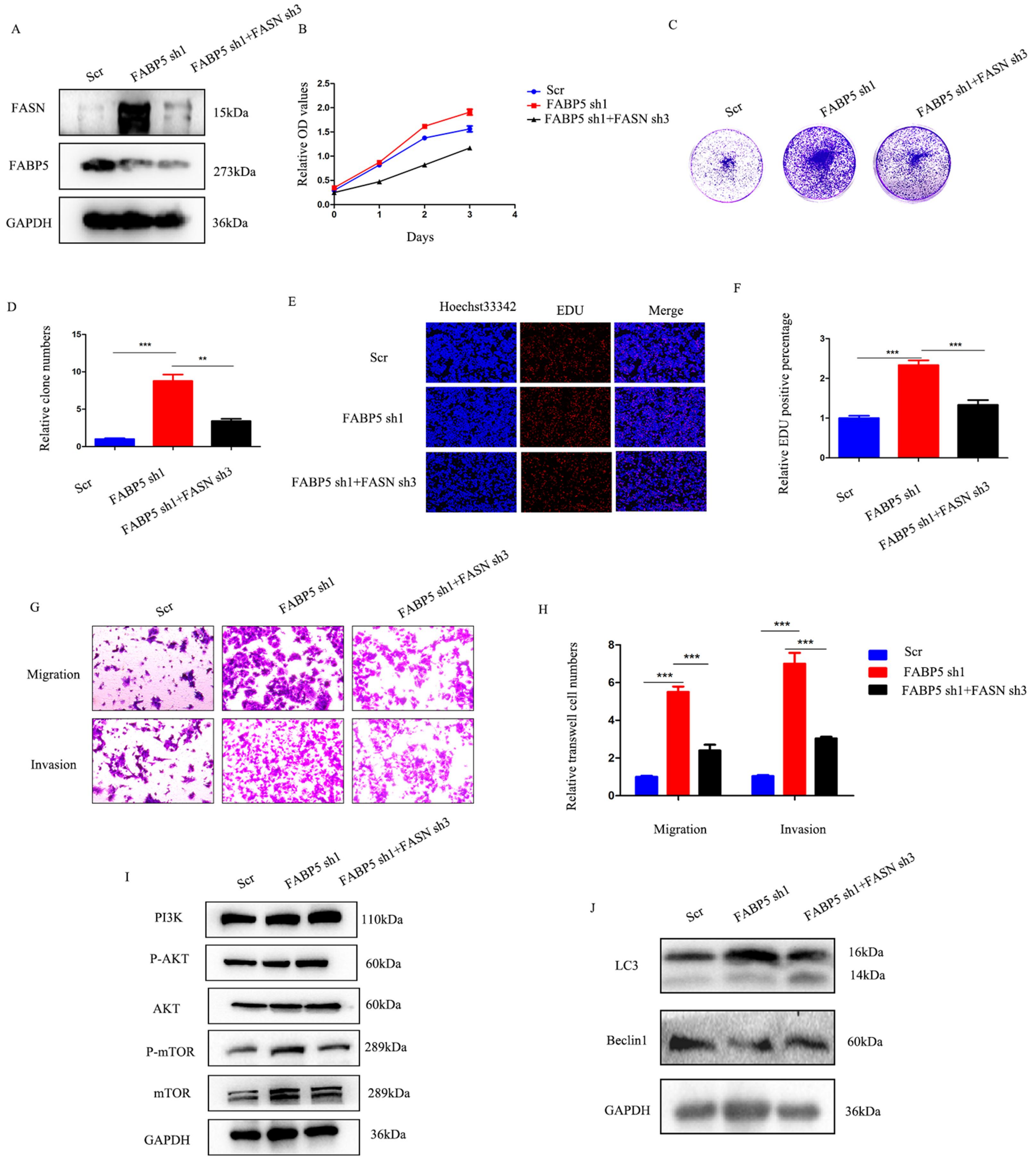

Supplementary Figure 3 (A, B) Enrichment analysis of transcriptome and lipid metabolomes in *FABP5* knockdown and control groups. (C, D) Western blot analysis of LC3/Beclin1 and PI3K/AKT/*mTOR* in HCT116 cells treated with 50 μmol/L orlistat for 24 h. (E, F) Western blot analysis of LC3/Beclin1 and PI3K/AKT/*mTOR* in *FASN* knockdown and control cells.

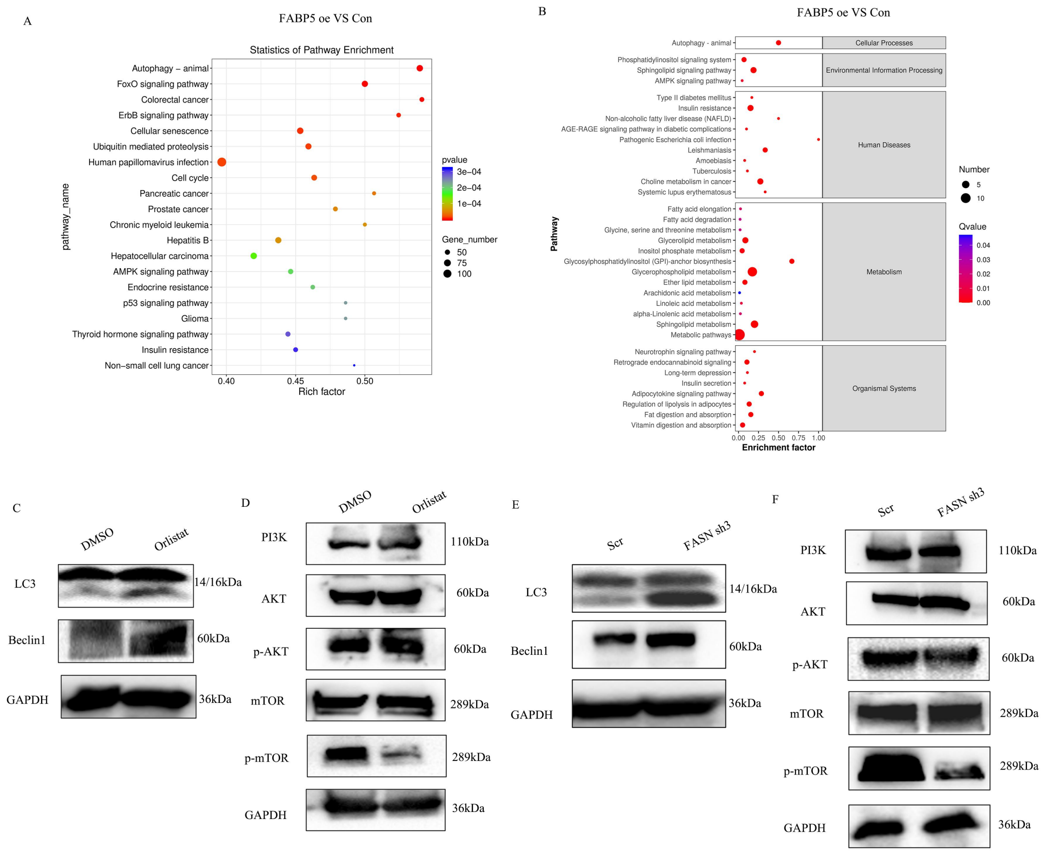

Supplement: Supplementary file 1 — Supplementary figures and tables. [file ijbsv19p3115s1.pdf]
